# KLU/CYP78A5, a Cytochrome P450 Monooxygenase Identified *via* Fox Hunting, Contributes to Cuticle Biosynthesis and Improves Various Abiotic Stress Tolerances

**DOI:** 10.3389/fpls.2022.904121

**Published:** 2022-06-23

**Authors:** Takuma Kajino, Masahiro Yamaguchi, Yoshimi Oshima, Akiyoshi Nakamura, Jumpei Narushima, Yukio Yaguchi, Izumi Yotsui, Yoichi Sakata, Teruaki Taji

**Affiliations:** ^1^Department of Bioscience, Tokyo University of Agriculture, Tokyo, Japan; ^2^Bioproduction Research Institute, National Institute of Advanced Industrial Science and Technology (AIST), Tsukuba, Japan; ^3^Electron Microscope Center, Tokyo University of Agriculture, Tokyo, Japan

**Keywords:** osmotic tolerance, heat tolerance, cuticular wax, CYP78A5, cuticle biosynthesis

## Abstract

Acquired osmotolerance after salt stress is widespread among *Arabidopsis thaliana* (Arabidopsis) accessions. Most salt-tolerant accessions exhibit acquired osmotolerance, whereas Col-0 does not. To identify genes that can confer acquired osmotolerance to Col-0 plants, we performed full-length cDNA overexpression (FOX) hunting using full-length cDNAs of halophyte *Eutrema salsugineum*, a close relative of Arabidopsis. We identified *EsCYP78A5* as a gene that can confer acquired osmotolerance to Col-0 wild-type (WT) plants. *EsCYP78A5* encodes a cytochrome P450 monooxygenase and the Arabidopsis ortholog is known as KLU. We also demonstrated that transgenic Col-0 plants overexpressing *AtKLU* (*AtKLU*ox) exhibited acquired osmotolerance. Interestingly, *KLU* overexpression improved not only acquired osmotolerance but also osmo-shock, salt-shock, oxidative, and heat-stress tolerances. Under normal conditions, the *AtKLU*ox plants showed growth retardation with shiny green leaves. The *AtKLU*ox plants also accumulated higher anthocyanin levels and developed denser cuticular wax than WT plants. Compared to WT plants, the *AtKLU*ox plants accumulated significantly higher levels of cutin monomers and very-long-chain fatty acids, which play an important role in the development of cuticular wax and membrane lipids. Endoplasmic reticulum (ER) stress induced by osmotic or heat stress was reduced in *AtKLU*ox plants compared to WT plants. These findings suggest that KLU is involved in the cuticle biosynthesis, accumulation of cuticular wax, and reduction of ER stress induced by abiotic stresses, leading to the observed abiotic stress tolerances.

## Introduction

Land plants grow under the influence of a wide range of biotic and abiotic stresses. The whole plant body is covered with a cuticle, an extracellular lipid structure acting as a protective barrier against these external stresses (Javelle et al., 2011; Delude et al., 2016). In particular, the plant cuticle plays an important role in limiting water loss (Riederer and Schreiber, 2001; Jenks et al., 2002; Aharoni et al., 2004; Kannangara et al., 2007). The cuticle is divided into a lower layer rich in cutin (cuticle layer) and an upper layer rich in wax (cuticle proper; Ingram and Nawrath, 2017). The cuticular wax is comprised of a mixture of mostly aliphatic very-long-chain fatty acid (VLCFA; C20–C34) derivatives, including alkanes, aldehydes, primary and secondary alcohols, ketones, and esters. VLCFAs are converted into aliphatic derivatives incorporated not only into the cuticle as cuticular waxes but also into another plant surface barrier, suberin, in root endodermis; into storage lipids as triacylglycerols; or into membrane lipids such as phospholipids or sphingolipids (Kim et al., 2013; Batsale et al., 2021). The defect in VLCFA biosynthesis causes not only a decrease in cuticular wax load, but also defects in endocytic membrane transport (Zheng et al., 2005). Thus, targeted manipulation of the biosynthetic pathway of cuticular wax could be a viable option for improving environmental stress tolerance in plants.

Cytochrome P450s (P450s) in plants play an important role in both primary metabolism and a wide variety of secondary metabolisms (Schuler and Werck-Reichhart, 2003). Several P450s are associated with fatty acid synthesis and cuticular wax production. In *Arabidopsis thaliana* (Arabidopsis), the *lacerata* (*lcr/cyp86a8*) mutant has a defect in the synthesis of epidermal cutin (Wellesen et al., 2001). The *att1* (*cyp86a2*) mutant of Arabidopsis reduces epidermal cutin content to 30% of that of wild-type (WT) plants and, unlike WT, is susceptible to *Pseudomonas syringae* (Xiao et al., 2004). The P450 proteins CYP78A5, CYP78A7, CYP78A10, and CYP86C3 can use lauric acid (C12), myristic acid (C14), palmitic acid (C16), and myristoleic acid (C14) as their substrates (Kai et al., 2009). CYP78A5, also known as KLU, is one of six members of the CYP78A family in Arabidopsis. While the *klu* mutant shows reduced growth of aerial organs, overexpression of *KLU* increases floral organ size; however, these phenotypes are not dependent on known phytohormones (Anastasiou et al., 2007; Cornet et al., 2021). Interestingly, *KLU*-overexpressing Arabidopsis not only shows resistance to various pathogens such as fungal pathogen *Rhizoctonia solani* and bacterial pathogen *P. syringae* pv. *tomato* DC3000 (Maeda et al., 2019), but also improves drought tolerance (Jiang et al., 2020). However, the mechanism underlying the biotic and abiotic stress tolerances of *KLU*-overexpressing plants has been unclear.

We previously found wide variation in salt tolerance among Arabidopsis accessions. Most salt-tolerant accessions, including Bu-5, exhibited acquired osmotolerance, whereas Col-0 did not (Katori et al., 2010). We later identified *ACQOS* as the locus responsible for this acquired osmotolerance (Ariga et al., 2017). However, little is known about how the tolerance is established. *Eutrema salsugineum* (formerly *Thellungiella halophila* or *T. salsuginea*) is closely related to Arabidopsis, and its genes show 90% identity to those of Arabidopsis. It is tolerant to extreme salinity stress as well as to chilling, freezing, ozone, and heat stress, suggesting that it is a good genomic resource for studies of tolerance to these abiotic stresses (Inan et al., 2004; Taji et al., 2004; Li et al., 2006; Higashi et al., 2013). We previously developed a full-length cDNA library of *E. salsugineum* derived from various tissues and whole seedlings subjected to environmental stress treatments (high salinity, chilling, and freezing) and abscisic acid (ABA) treatment (Taji et al., 2008, 2010). We then generated full-length cDNA overexpressing (FOX) plasmids by introducing each cDNA into a binary vector downstream of the CaMV 35S promoter, and produced many Arabidopsis Col-0 transgenic lines (FOX lines) transformed independently with each FOX plasmid (Higashi et al., 2013; Ariga et al., 2015). Here, we performed “FOX hunting,” a high-throughput strategy to analyze the physiological functions of genes (Ichikawa et al., 2006), as a way to identify *E. salsugineum* genes that could confer acquired osmotolerance to Arabidopsis Col-0. We identified a candidate gene encoding CYP78A5 (KLU) and explored the mechanisms by which this gene promotes osmotolerance.

## Materials and Methods

### Plant Materials and Growth Conditions

*Arabidopsis thaliana* seeds (Col-0) were sown on agar [0.8% (w/v)] plates containing full-strength Murashige and Skoog (MS) salts with a vitamin mixture (10 mg l^−1^ myoinositol, 200 μg l^−1^ glycine, 50 μg l^−1^ nicotinic acid, 50 μg l^−1^ pyridoxine hydrochloride, 10 μg l^−1^ thiamine hydrochloride, pH 5.7) and 1% (w/v) sucrose. Plates were sealed with surgical tape. The seeds were stratified at 4°C for 4–7 days and then transferred to a growth chamber (80 μmol photons m^−2^ s^−1^; 16 h/8 h light/dark cycle; 22°C) for germination and growth. The production of FOX Arabidopsis lines in a Col-0 genetic background was described in Higashi et al. (2013).

### Fox Hunting

To screen the FOX lines for acquired osmotolerance, salt-acclimated 2-week-old seedlings were mesh-transferred to MS agar plates containing 750 mM sorbitol. We considered a gene as a candidate if two or more independent transgenic lines containing that gene were significantly more tolerant than WT plants. Since not many seeds were obtained for the *EsKLU*ox line, a candidate for the FOX line, the *AtKLU*ox, the Arabidopsis *KLU* homolog lines were used in subsequent experiments.

### Generation of *AtKLU*ox Plants

The cDNA region of *AtKLU* was amplified by PCR with pGH_AtCYP78A5/KLU primers (Supplementary Table S1) and cloned into the binary vector pGreen0029 downstream of the 35S promoter. The constructs were introduced into *Agrobacterium tumefaciens* GV3101, which was used for plant transformation by the floral dip method. Transgenic plants were selected on MS agar plates containing 200 μg ml^−1^ Claforan and 25 μg ml^−1^ kanamycin. Ten-day-old seedlings (T1 plants) were transferred to soil in pots.

### Stress Treatment for the Acquired Osmotolerance Assay

Seven-day-old seedlings (WT and *EsKLU*ox or *AtKLU*ox) grown on nylon mesh (990 μm) on an MS agar plate were mesh-transferred to a plate supplemented with 100 mM NaCl for 7 days. The seedlings were then mesh-transferred to a plate supplemented with 750 mM sorbitol for 15 days.

### Abiotic Stress Assays

Ten-day-old WT and *AtKLU*ox seedlings grown on nylon mesh (990 μm) on an MS agar plate were mesh-transferred to a plate supplemented with 200 mM NaCl for 7 days (salt-shock stress), 750 mM sorbitol for 21 days (osmo-shock stress), or 10 μm paraquat for 14 days (oxidative stress). The L-heat (long-term) and S-heat (short-term) heat stress tolerance assays were performed as described in Isono et al. (2020). Chlorophyll content was determined as in Porra et al. (1989).

### RNA Extraction and qRT-PCR

Total RNA extraction and qRT-PCR were performed as described in Isono et al. (2020). *Actin2* was used as an internal standard for qRT-PCR. Primers are listed in Supplementary Table S2.

### Determination of Anthocyanin Content

The aerial parts of 2-week-old WT and *AtKLU*ox seedlings were harvested for anthocyanin measurement using the method described by Rabino and Mancinelli (1986).

### Toluidine Blue Test

The aerial parts of 2-week-old WT and *AtKLU*ox seedlings were submerged in aqueous solution of 0.05% (w/v) toluidine blue (TB; Sigma, St Louis, MO, United States of America). After 20 min on a shaker set at 100 rpm, the TB solution was removed, and plates were washed gently with water to remove excess TB from the plants. The plants were homogenized in 1.5-ml tubes containing zirconia beads. Next, 200 μl of buffer [200 mM Tris–HCl (pH 8.0), 250 mM NaCl, 25 mM EDTA] and 400 μl of ethanol was added, with vortex mixing, and the plant debris was pelleted by centrifugation. The supernatant was examined spectrophotometrically, and the amount of TB was determined from the absorbance at 630 nm (A630). The major peak of absorbance due to plant material (A435) was used for normalization. Relative levels of TB were calculated as the ratio of A630:A435 (Tanaka et al., 2007).

### Scanning Electron Microscopy

A 3-cm section from the base of the flower stem of WT or *AtKLU*ox 2 weeks after bolting was used for observation of the epidermal surface. Each stem was cut into 5 ~ 10 mm pieces and coated with Pt + Pd using an E102 ion sputter (Hitachi, Japan) for scanning electron microscopy (S4800; Hitachi, Japan).

### Water Loss Assay

Vaseline (Daiwa Chemical, Osaka, Japan) was applied to the underside of leaves of 4-week-old WT and *AtKLU*ox plants grown in soil under normal growth conditions to reduce evaporation from stomata. The aerial parts were then detached and left under ambient conditions. The weight was measured every 10 min for 60 min. The percentages of decrease in fresh weight were expressed as percentage of water loss.

### Extraction of Cuticular Waxes and Gas Chromatography–Mass Spectrometry Analysis

Cuticular wax of leaves from simultaneously grown 5-week-old WT and *AtKLU*ox plants, with six biological replicates, was extracted by immersing the leaves for 10 s in chloroform containing tricosanoic acid as an internal standard. The solvent was evaporated in a stream of nitrogen. Free hydroxyl and carboxyl groups were silylated with *N*,*O*-bis(trimethylsilyl)trifluoroacetamide (BSTFA + TMCS; Sigma-Aldrich, St. Louis, MO, United States of America) for 1 h at 80°C. The wax composition was analyzed by GC2020 gas chromatography (Shimadzu Inc., Kyoto, Japan) with the injector in splitless mode with the temperature programed starting at 80°C, increasing 15–200°C/min, then increasing 3–300°C/min, followed by a 10-min hold at 300°C. Mass spectrum data were obtained on a GCMS-QP2020NX mass spectrometer (Shimadzu) after impact ionization. The peaks were quantified using the Gas Chromatography–Mass Spectrometry (GC–MS) LabSolutions software (Shimadzu). Each wax monomer amount was determined on the basis of the internal standard and normalized by sampled leaf surface area. Leaf surface area was calculated by ImageJ software (Schneider et al., 2012).

### Lipid Polyester Monomer Analysis

For polyester analysis, 300 mg of leaves from simultaneously grown plants with six biological replicates were used. Polyester extraction and analysis were performed by the method described previously with slight modification (Delude et al., 2017). Whole leaves were delipidated and followed by methanolysis with sulfuric acid in methanol. Methyl heptadecanoate and ω-pentadecalactone (Tokyo Chemical Industry Co., Ltd., Japan) were added as internal standards. Depolymerized compounds were silylated with BSTFA + TMCS (Sigma-Aldrich Inc.) for 20 min at 100°C and analyzed by GC–MS (GCMS-QP2020NX; Shimadzu Inc.) with a Rtx-5 column (Restek Corporation PA, United States of America) at the temperature programed from 50°C, 25°C/min to 200°C, followed by a 1 min hold, and increased at a rate of 10°C/min to 320°C and an 8 min hold. The mass spectrum data were analyzed as mentioned above.

## Results

### FOX Hunting for Genes Conferring Acquired Osmotolerance

**Figure 1:**
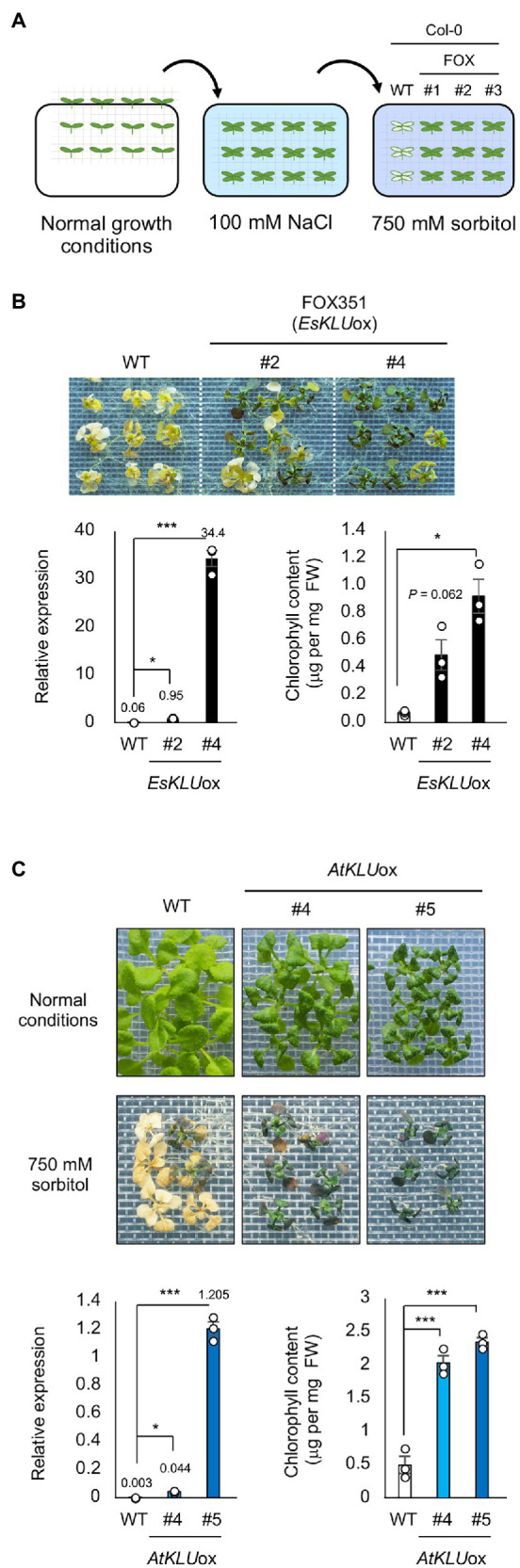
Acquired osmotolerance of *EsKLUox* and *AtKLU*ox plants. **(A)** Flowchart of the acquired osmotolerance assay. Salt-acclimated 2-week-old seedlings of WT and FOX lines were mesh-transferred to Murashige and FIGURE 1 | Skoog (MS) agar plates containing 750 mM sorbitol for 15 days. **(B)** Top panel: Acquired osmotolerance of *EsKLU*ox plants. #2 and #4 are independent FOX351 lines. Lower left panel: Relative expression of *AtKLU* or *EsKLU* in WT or *EsKLU*ox plants, respectively, under normal conditions; expression levels were determined by quantitative real-time PCR relative to those of *Actin2* (mean ± SE, *n* = 3). The numbers on the bars are the relative expressions. Lower right panel: Chlorophyll content of WT and *EsKLU*ox plants following treatment on NaCl and sorbitol as described in (top). **(C)** Top panel: Two-week-old WT and *AtKLU*ox plants under normal growth conditions (top row) and on 750 mM sorbitol following treatment on 100 mM NaCl (bottom). #4 and #5 are independent *AtKLU*ox lines. Lower left panel: Relative expression of *AtKLU* in WT and *AtKLU*ox plants under normal conditions; expression levels were determined by quantitative real-time PCR relative to those of *Actin2* (mean ± SE, *n* = 3). The numbers on the bars are the relative expressions. Lower right panel: Chlorophyll content of WT and *AtKLU*ox plants following treatment on NaCl and sorbitol as described in (top). Differences between WT and *EsKLU*ox or *AtKLU*ox plants were analyzed by Student’s *t*-test (mean ± SE, *n* = 3, ^*^P < 0.05, ^***^P < 0.001).

To screen the FOX lines for acquired osmotolerance, salt-acclimated 2-week-old seedlings were mesh-transferred to MS agar plates containing 750 mM sorbitol. We considered a gene as a candidate if two or more independent transgenic lines containing that gene were significantly more tolerant than WT plants (Figure 1A). FOX351 plants showed distinct acquired osmotolerance (Figure 1B; Supplementary Figure S1). The transgene in FOX351 lines encodes the cytochrome P450 monooxygenase that shows the highest sequence identity to Arabidopsis CYP78A5, also known as KLU, one of six members of the CYP78A family in Arabidopsis. We generated more than 5 independent EsKLUox lines (Supplementary Figure S1). Two independent lines, FOX351 (*EsKLU*ox) #2 and #4, showed significantly higher expression of *KLU* (presumably encoded by the transgene) than WT plants (Figure 1B). It has been reported that *KLU*-overexpressing Arabidopsis plants have twisted flower stems, short pods, and extremely reduced fertility (Zondlo and Irish, 1999). As in this previous report, the flower stems of both *EsKLU*ox lines exhibited a twisted, zigzag appearance, and the fertility of both *EsKLU*ox lines was greatly reduced (Supplementary Figure S2A). Therefore, these *EsKLU*ox lines were not used for further analysis.

To determine whether transgenic plants overexpressing *AtKLU*, the Arabidopsis *KLU* homolog, would show greater acquired osmotolerance than WT plants, we produced more than four independent *AtKLU*ox lines *AtKLU*-overexpressing (*AtKLU*ox) Arabidopsis Col-0 plants. Under normal growth conditions, *AtKLU*ox plants showed growth retardation in a *KLU* expression level–dependent manner (Figure 1C). Two independent *AtKLU*ox lines exhibited greater acquired osmotolerance than WT plants, similar to that of *EsKLU*ox plants (Figure 1C; Supplementary Figure S1). Similar to *EsKLU*ox plants, *AtKLU*ox plants displayed growth retardation, twisted flower stems, and reduced fertility (Supplementary Figures S2A,B).

### *AtKLU*ox Plants Exhibit Various Abiotic Stress Tolerances

**Figure 2:**
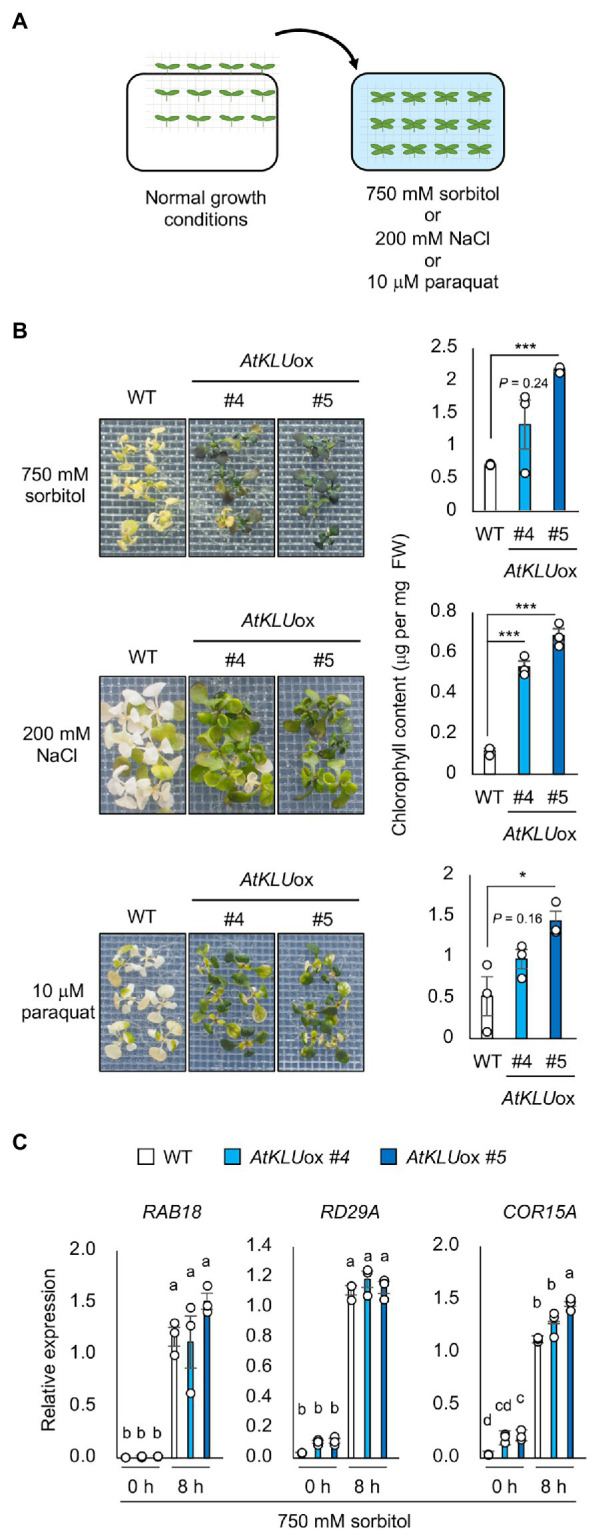
Osmo-shock, salt-shock, and oxidative tolerance of *AtKLU*ox plants. **(A)** Flowchart of the osmo-shock, salt-shock, and oxidative tolerance assays. **(B)** Osmo-shock, salt-shock, and oxidative stress tolerances of *AtKLU*ox plants. Ten-day-old seedlings were mesh-transferred to MS agar plates containing 750 mM sorbitol for 21 days, 200 mM NaCl for 7 days, or 10 μM paraquat (oxidative stress inducer) for 14 days. Right panel: Chlorophyll content of seedlings shown at left. Differences between WT and *AtKLU*ox plants were analyzed by Student’s *t*-test (mean ± SE, *n* = 3, ^*^p < 0.05, FIGURE 2 | ^***^p < 0.001). **(C)** Expression profiles of osmo-responsive marker genes in WT and *AtKLU*ox plants under normal (0 h) and acquired osmotic stress (100 mM NaCl for 7 days followed by 750 mM sorbitol for 8 h) conditions; expression levels were determined by quantitative real-time PCR relative to those of *Actin2*. Bars labeled with different letters differ significantly (*P* < 0.05, one-way ANOVA with *post hoc* Tukey HSD test, mean ± SE, *n* = 3).

To further characterize the *AtKLU*ox plants, we examined their tolerance to other abiotic stresses. The *AtKLU*ox plants exhibited not only acquired osmotolerance but also osmo-shock, salt-shock and oxidative stress tolerance compared with WT plants (Figures 2A,B). To investigate the osmotic stress response in *AtKLU*ox plants at the transcriptional level, we examined the expression profiles of three osmotic response marker genes: *RAB18*, *RD29A*, and *COR15A*. Expression of all three genes increased under osmotic stress in both *AtKLU*ox and WT plants. Their expression levels were comparable in WT and *AtKLU*ox plants, except for that of *COR15A* in *AtKLU*ox_#5 plants, which was significantly higher than in WT (Figure 2C).

**Figure 3:**
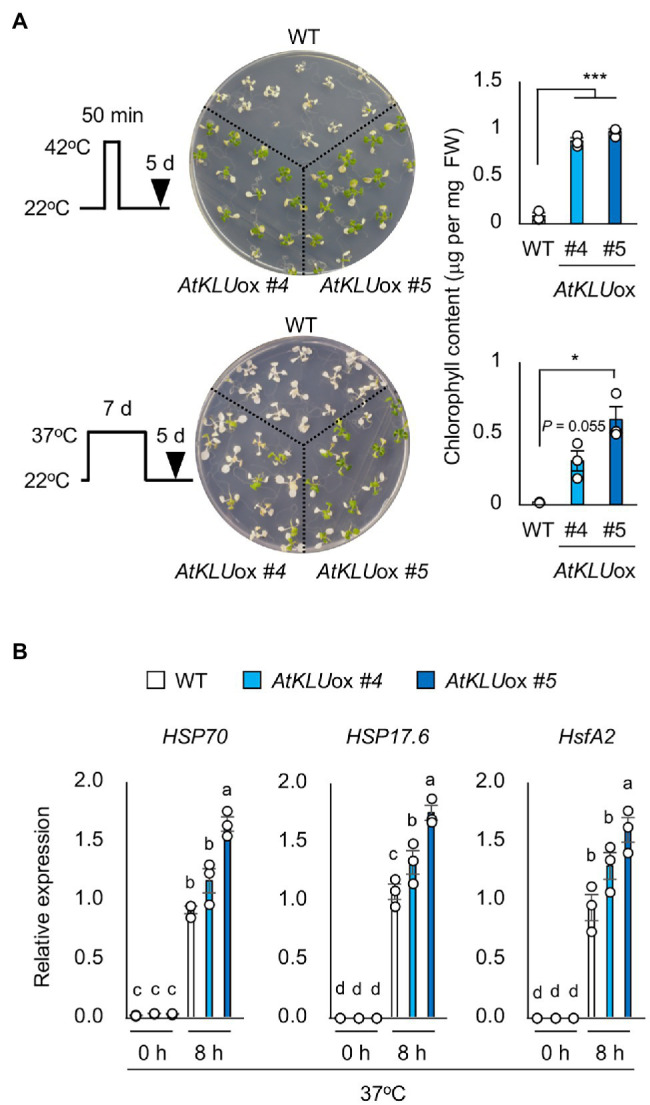
Heat stress tolerance of *AtKLU*ox plants. **(A)** Upper left panel: Flowchart of S-heat tolerance assay. Upper middle panel: S-heat tolerance of WT and *AtKLU*ox plants. Ten-day-old seedlings grown at 22°C (normal conditions) were placed at 42°C for 50 min and then grown at 22°C for 5 days. Upper right panel: Chlorophyll content of seedlings shown at left. Lower left panel: Flow of L-heat tolerance assay. Lower middle panel: 10-day-old WT and *AtKLU*ox seedlings grown at 22°C were placed at 37°C for 5 days and then grown at 22°C for 5 days. Lower right panel: Chlorophyll content of seedlings shown in left. Differences between WT and *AtKLU*ox plants were analyzed by Student’s *t*-test (mean ± SE, *n* = 3, ^*^P < 0.05, ^***^P < 0.001). **(B)** Expression of *HSP70*, *HSP17.6*, and *HsfA2* in WT and *AtKLU*ox plants under normal (0 h) and heat stress (37°C for 8 h) conditions; expression levels were determined by quantitative real-time PCR relative to those of *Actin2*. Bars labeled with different letters differ significantly (*P* < 0.05, one-way ANOVA with *post hoc* Tukey HSD test, mean ± SE, *n* = 3).

In addition, we evaluated the tolerance of *AtKLU*ox plants against long-term (L-) heat stress (37°C, 5 days) or short-term (S-) heat stress (42°C, 50 min). The tolerance of *AtKLU*ox plants to both L- and S-heat stresses was significantly higher than that of WT plants (Figure 3A). We investigated the expression profiles of three heat response marker genes: *HSP70*, *HSP17.6*, and *HsfA2*. The induction levels were almost comparable between WT and *AtKLU*ox plants, but the transcript levels under heat stress tended to be higher in *AtKLU*ox, especially in *AtKLU*ox_#5 plants, than in WT (Figure 3B).

### KLU Plays a Role in Development of Epicuticular Wax

**Figure 4:**
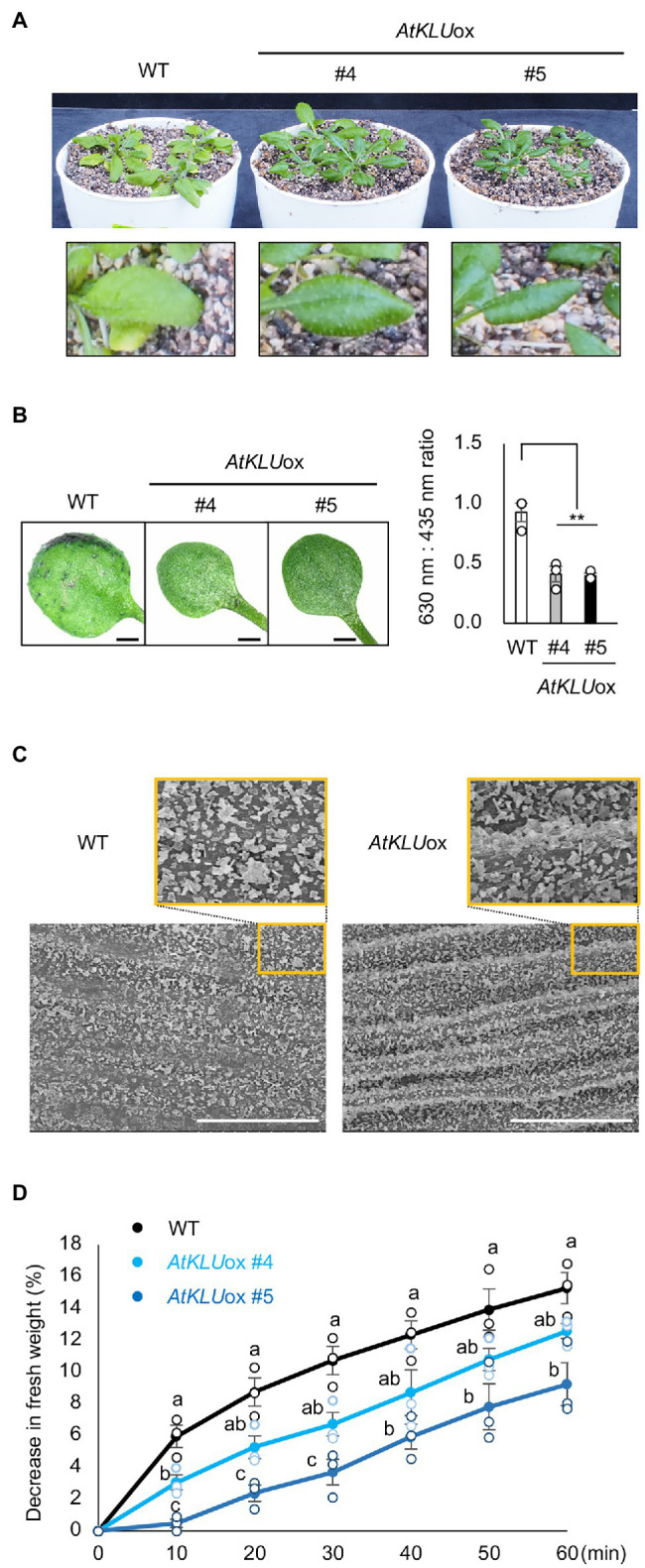
Epidermal cuticular wax of *AtKLU*ox plants. **(A)** Four-week-old WT and *AtKLU*ox plants grown in soil under normal conditions. Lower panel: Magnified view of their leaves. **(B)** Toluidine blue (TB) test. Left panel: Two-week-old WT and *AtKLU*ox plants stained with TB. Right panel: TB extract was examined spectrophotometrically, and the amount of TB was determined by the absorbance at 630 nm (A630). The major peak of absorbance due to FIGURE 4 | plant material (A435) was used for normalization. Relative levels of TB were calculated as the ratio of A630:A435. Differences between WT and *AtKLU*ox plants were analyzed by Student’s *t*-test (mean ± SE, *n* = 3, ^**^P < 0.01). **(C)** Stem surface of WT and *AtKLU*ox #5 observed by scanning electron microscopy. Bars = 100 mm. **(D)** Water loss from detached WT and *AtKLU*ox leaves. The entire aerial parts of 4-week-old plants grown in soil under normal growth conditions were detached (0 min) and then left under ambient conditions for 60 min, with measurements taken every 10 min. The percentage decrease in fresh weight was used as the percentage water loss. Bars labeled with different letters differ significantly (*P* < 0.05, one-way ANOVA with *post hoc* Tukey HSD test, mean ± SE, *n* = 3).

The leaves of *AtKLU*ox plants were darker green under normal growth conditions than those of WT (Figure 1C). We found that *AtKLU*ox plants accumulated higher amounts of anthocyanins than WT plants (Supplementary Figure S3). Moreover, *AtKLU*ox plants grown in soil under normal conditions displayed a shiny green leaf surface (Figure 4A), which is a known phenotype when cuticular wax is increased (Aharoni et al., 2004; Sajeevan et al., 2017). The TB test was established for the detection of cuticular defects in whole leaves, and a deficient cuticle allows TB to permeate the epidermal surface (Tanaka et al., 2004). Leaves of WT plants were partially stained by TB, whereas those of *AtKLU*ox plants showed almost no staining, suggesting an increase in cuticular wax on the surface of *AtKLU*ox leaves (Figure 4B). To investigate the morphology of the cuticular wax, we observed epicuticular wax crystals on the stem of WT and *AtKLU*ox plants by scanning electron microscopy. Both plant types showed large numbers of granular wax crystals on the stem surface, but the stem of *AtKLU*ox plants was more densely covered than that of WT plants (Figure 4C). These findings suggest that KLU has an important role in the development of epicuticular wax crystals.

It was previously reported that *KLU*-overexpressing Arabidopsis plants showed enhanced drought tolerance and reduced water loss, which was due to the slight stomatal closure (Jiang et al., 2020). On the other hand, increased cuticular wax is known to suppress water loss from the leaf surface and improve drought tolerance (Aharoni et al., 2004). It is known that more water was lost through abaxial surface than that of adaxial surface, and vaseline application can effectively reduce leaf water loss (Zhang et al., 2020). We tested the drought tolerance and water loss of detached whole aerial parts after applying vaseline to the underside of the leaves to reduce the evaporation from stomata. The *AtKLU*ox plants showed reduced water loss compared to WT plants (Figure 4D). These results suggest that KLU plays an important role in drought tolerance by inhibiting water loss from the leaf surface.

### KLU Contributes to Cuticle Biosynthesis

**Figure 5:**
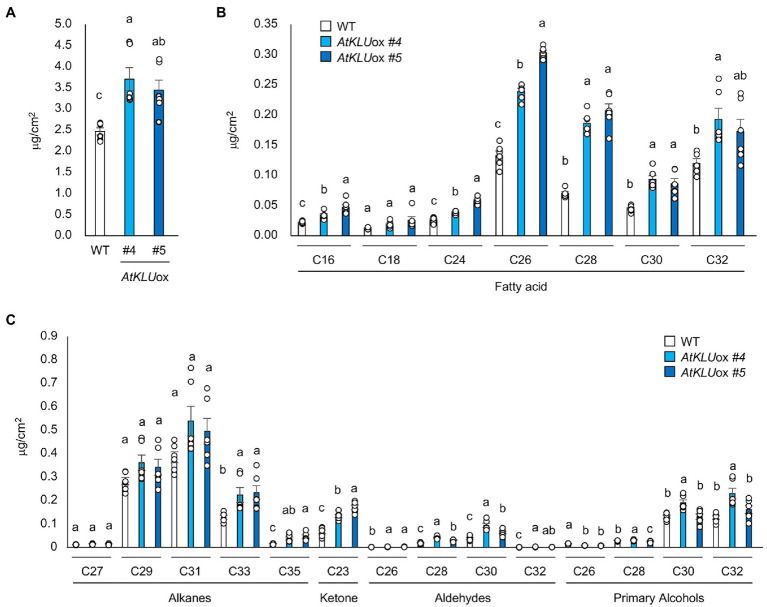
Cuticular wax content and composition of 4-week-old WT and *AtKLU*ox seedlings. **(A)** Total wax content of WT and *AtKLU*ox plants. **(B)** Fatty acid (C16–C32) contents in WT and *AtKLU*ox plants. **(C)** Identified waxes (alkanes, ketones, aldehydes, and primary alcohols) in WT and *AtKLU*ox plants. Data represent means ± SE, *n* = 6. Within each compound type and chain length, values marked with the same letter are not significantly different based on one-way ANOVA and Tukey’s test, *P* < 0.05.

To investigate the biochemical basis of the increased number of epicuticular wax crystals observed on *AtKLU*ox plants, we determined the wax components including fatty acids, primary alcohols, aldehydes, and alkanes on the leaf surface of WT and *AtKLU*ox plants grown under normal growth conditions. The *AtKLU*ox plants showed significantly higher accumulation of most fatty acids (C16–C32) than did WT plants, but notably, the highest levels (and the largest differences) were for VLCFAs with chain length ≥ C26 (Figures 5A,B). VLCFAs are precursors of all the aliphatic components of cuticular wax, and plant cuticular VLCFAs and derivatives generally have around 30 carbon atoms (Hegebarth et al., 2016; Batsale et al., 2021). One or both *AtKLU*ox lines also had significantly higher levels of alkanes with chain length ≥ C33, ketone (C23), aldehydes, and primary alcohols with chain length ≥ C28 than WT plants (Figure 5C; Supplementary Figure S4).

**Figure 6:**
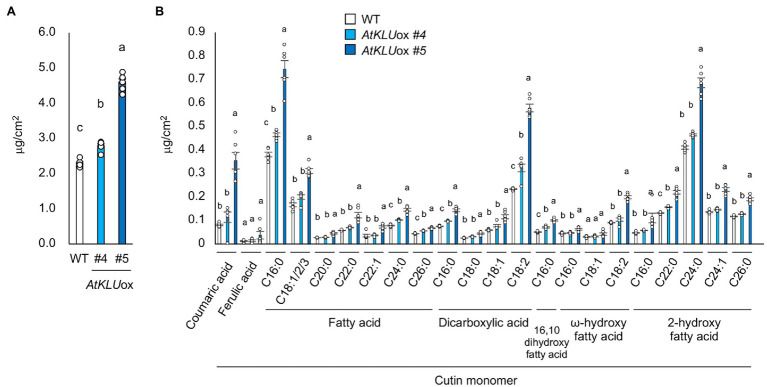
Cutin monomer content and composition of 4-week-old WT and *AtKLU*ox seedlings. **(A)** Total cutin monomer content of WT and *AtKLU*ox plants. **(B)** Identified cutin monomers (coumaric acid, ferulic acid, fatty acids, dicarboxylic acids, 16, 10 dihydroxy fatty acid, ω-hydroxy fatty acids, and 2-hydroxy fatty acids) in WT and *AtKLU*ox plants. The C16 ~ 26 labels on the x axis indicate chain length. Data represent means ± SE, *n* = 6. Within each compound type and chain length, values marked with the same letter are not significantly different based on one-way ANOVA and Tukey’s test, *P* < 0.05.

To further investigate the function of KLU in cuticular lipid biosynthesis, we quantified cutin monomers including coumaric acid, ferulic acid, fatty acids, dicarboxylic acids, 16, 10 dihydroxy fatty acid, ω-hydroxy fatty acids, and 2-hydroxy fatty acids on the leaf surface of WT and *AtKLU*ox plants grown under normal growth conditions. The *AtKLU*ox plants exhibited significantly higher accumulation of most cutin monomers than did WT plants (Figures 6A,B). These findings suggest that KLU contributes to cuticle biosynthesis.

### ER Stress Is Suppressed in *AtKLU*ox Plants Under Osmotic Stress

**Figure 7:**
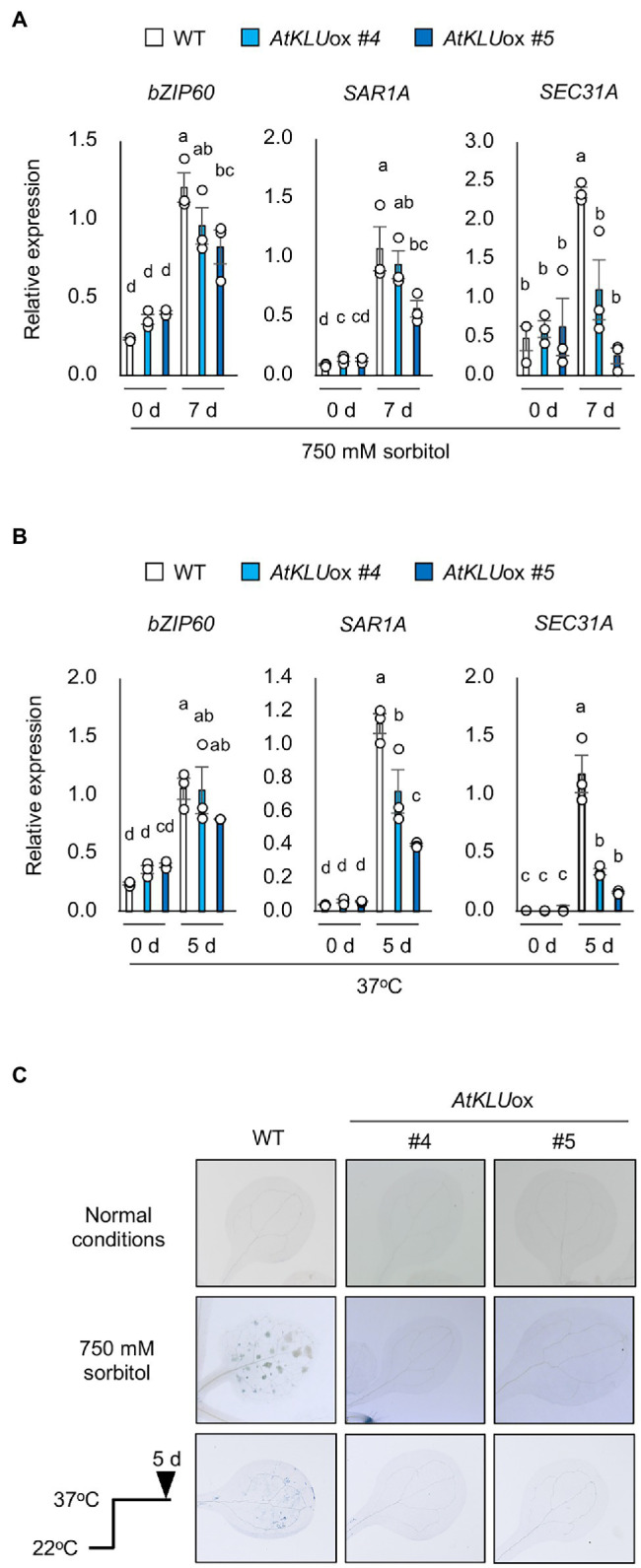
ER stress in *AtKLU*ox plants under osmotic or heat stress. **(A)** Transcript levels of *bZIP60*, *SAR1A*, and *SEC31A* in WT and *AtKLU*ox plants under normal (0 day) and osmotic stress condition (750 mM sorbitol for 7 days). **(B)** Transcript levels of *bZIP60*, *SAR1A*, and *SEC31A* in WT and *AtKLU*ox plants under normal (0 day) and heat stress (37°C for 8 h) conditions. Expression levels were determined by quantitative real-time PCR relative to FIGURE 7 | those of *Actin2*. Bars labeled with different letters differ significantly (*P* < 0.05, one-way ANOVA with *post hoc* Tukey HSD test, mean ± SE, *n* = 3). **(C)** Trypan blue staining of leaves of WT or *AtKLU*ox plants under normal, osmotic stress (750 mM sorbitol for 7 days), and heat stress (37°C for 5 days) conditions.

An Arabidopsis enoyl-coenzyme A reductase, *ECERIFERUM 10* (*CER10*) is involved in the elongation reactions of VLCFAs. The Arabidopsis *cer10* mutant is reported to show a reduction of cuticular wax load and altered VLCFA composition of seed triacylglycerols and sphingolipids; in addition, the Golgi apparatus is larger and tends to form ring-like clusters, resulting in a possible defect in endocytic membrane transport (Zheng et al., 2005; Batsale et al., 2021). The defect in ER-to-Golgi transport impairs the salt and L-heat tolerances of Arabidopsis plants (Isono et al., 2020). Environmental stress such as high temperature induces the accumulation of misfolded or unfolded proteins in the ER (Liu and Howell, 2010; Isono et al., 2020). ER stress evokes the unfolded protein response (UPR), which lightens the load of such proteins through increased ER chaperone production that aids protein folding (Howell, 2013). bZIP60 is a major activator of the canonical UPR, and genes regulated by bZIP60 have been identified (Kim et al., 2018). To investigate whether ER stress is reduced under osmotic stress in *AtKLU*ox plants, we examined the transcript levels of *bZIP60* and two of its regulated genes, *SAR1A* and *SEC31A*, in WT and *AtKLU*ox plants under osmotic or heat stress. Expression of all three genes was increased by osmotic or heat stress in both WT and *AtKLU*ox plants, but the transcript levels of *SAR1A* and *SEC31A* were significantly lower in *AtKLU*ox plants than in WT (Figures 7A,B). This result suggests that osmotic and heat stresses induce ER stress *via* bZIP60, but that ER stress was reduced in *AtKLU*ox plants relative to WT as evidenced by the reduced levels of *SAR1A* and *SEC31A* expression.

It is known that ER stress leads to programmed cell death under severe or long-term stress (Watanabe and Lam, 2008; Howell, 2013). Therefore, WT and *AtKLU*ox plants were exposed to osmotic or heat stress, and cell death was assessed by trypan blue staining. The results showed that cell death was suppressed under both stresses in *AtKLU*ox plants compared to WT plants (Figure 7C).

### KLU May Be Able to Use Long-Chain Fatty Acids as Substrates

**Figure 8:**
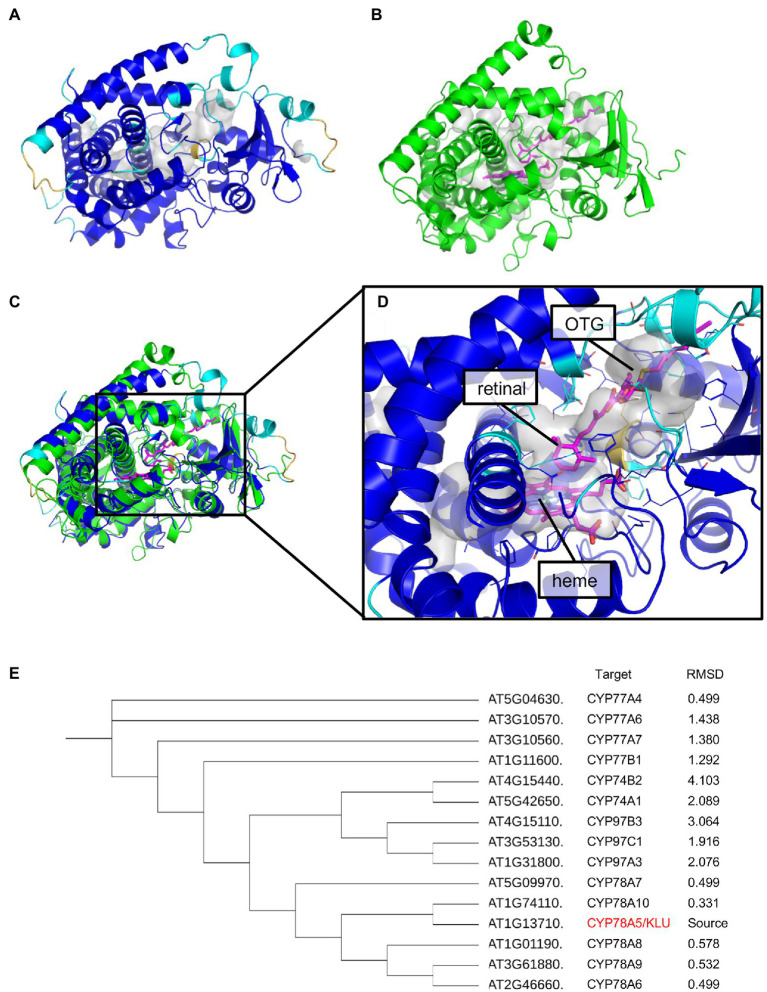
Protein structure prediction of AtKLU. **(A)** The predicted structure of CYP78A5 colored according to the pLDDT values [blue (high) to yellow (low)]. The internal cavity of CYP78A5 is shown as a gray surface. **(B)** The crystal structure of CYP97C1 (green) with heme and octylthioglucoside (OTG; magenta). The location of retinal (magenta) was modeled by superposing the CYP97A3-retinal complex (PDB ID: 6L8J) onto CYP97C1 (PDB ID: 6L8H). The putative lutein-binding cavity in CYP97C1 is shown as a gray surface. **(C)** Superposition of CYP78A5 and CYP97C1. **(D)** The predicted active site of CYP78A5 [enlarged from **(C)**]. The residues involved in constructing the internal cavity are shown as line models. The location of heme, OTG, and retinal (magenta) were modeled by superposing CYP97C1 onto the CYP78A5 model. **(E)** Phylogenetic tree created from amino acid sequences of CYP-74, -77, -78, and -97 with root mean square deviation (RMSD) values.

The substrates of KLU/CYP78A5 are currently unclear. Since KLU does not appear to modulate the levels of known phytohormones, it has been suggested that KLU is involved in generating a mobile growth signal distinct from the classical phytohormones (Anastasiou et al., 2007; Jiang et al., 2020). VLCFA are elongated from C16 and C18 long-chain fatty acids synthesized in the plastid by the fatty acid synthase (FAS) complex (Batsale et al., 2021). The present study revealed a high accumulation of cutin monomers and VLCFA derivatives in *KLU*-overexpressing plants, suggesting that KLU may function as a member of the FAS complex. However, it is unclear whether KLU directly or indirectly contributes to the biosynthesis of these cuticular wax components. Very recently, AlphaFold2 was developed as a computational method that can regularly predict three-dimensional (3D) protein structures with atomic accuracy (Jumper et al., 2021). When we predicted the protein structure of KLU with AlphaFold2, the 3D model showed a high pLDDT value except for the N-terminus of the membrane binding site, which indicates that the predicted 3D model is reliable (Figure 8A). Then, we searched for a structural homolog of the predicted structure without the N-terminus of KLU by using PDBeFold (Krissinel and Henrick, 2004). The top hit (*Z* score = 15.1, Root Mean Square Deviation [RMSD] = 1.95 Å [399 aa]) was Arabidopsis CYP97C1 (ID: 6L8H; Figure 8B). CYP97C1 and CYP97A3 catalyze hydroxylation of the β- and e-rings of α-carotene to produce lutein (Quinlan et al., 2012). The crystal structures of retinal-bound CYP97A3 and CYP97C1 in a detergent (octylthioglucoside; OTG)-bound form have been determined (Niu et al., 2020).

Based on the superposition of KLU/CYP78A5 and CYP97C1 (Figure 8C) and the conservation of P450s, the predicted 3D model of KLU has an internal cavity that we assumed to be partly a binding site for heme (Figure 8D). Similar to CYP97C1, KLU/CYP78A5 has a long cavity (~ 18 Å) in the direction of the predicted binding site of lutein in CYP97C1. Previous biochemical experiments have suggested that KLU/CYP78A5 catalyzes the hydroxylation of short-chain fatty acids such as lauric acid, although lauric acid might not be the substrate *in planta* because an application of lauric acid could not complement the *klu/cyp78a5* mutant phenotype (Kai et al., 2009). The cavity adjacent to the heme-binding site has sufficient space for retinal or lauric acid (which are similar in size) and may also allow the binding of longer-chain fatty acids.

CYP77A6, which shows high similarity to KLU at the amino acid sequence level, has been suggested to contribute to the production of dihydroxy fatty acids that compose cutin (Oshima and Mitsuda, 2016). To investigate the homology between KLU/CYP78A5 and CYP77A6, we constructed a phylogenetic tree of CYP-78, -77, -74, and -97 proteins and compared their 3D structure models using the PyMOL molecular visualization program. In the phylogenetic tree, the CYP78 proteins were closest to each other, followed by CYP-97, -74, and -77 (Figure 8E). On the other hand, in the comparison of RMSD values (which indicate 3D similarity), the closest values were between the CYP78 proteins, followed by CYP-77, -97, and -74. Taken together, these data suggest that KLU/CYP78A5 may be able to use long-chain fatty acids as substrates, although further biochemical and structural analysis are needed.

## Discussion

We found that *KLU* overexpression enhanced tolerance to a wide range of stresses, including osmotic, salt, oxidative, S-heat, and L-heat stresses. *AtKLU*ox plants were densely covered with granular wax crystals and had high accumulation of cutin monomers and VLCFAs compared to WT. The cuticle is strategically located at the plant–air interface and is a major player in plant protection against environmental stressors such as drought, UV radiation, and pathogen or insect attacks (Shepherd and Griffiths, 2006). The epidermal cuticular wax inhibits not only water loss induced by drought stress, but also entry of pathogens (Wang et al., 2020). *KLU*-overexpressing Arabidopsis plants exhibit resistance to both fungal and bacterial pathogens (Maeda et al., 2019). The mechanism of this resistance is unknown, but may be because the accumulation of cuticular wax prevents the entry of pathogens. In addition, *AtKLU*ox plants accumulated large amounts of anthocyanin under normal growth conditions. Although it remains unclear whether anthocyanin accumulation is due to direct or indirect effects of *KLU* overexpression, anthocyanin accumulation may also enhance abiotic stress tolerance.

*KLU* overexpression enhances tolerance to a wide range of environmental stresses, and therefore may be very useful in breeding strategies for stress-tolerant crops. However, *AtKLU*ox plants exhibited growth retardation, twisted flower stems, and reduced fertility. Since these phenotypes are dependent on the expression level, it will be necessary to determine whether *KLU* can be expressed in crop plants at a level sufficient to enhance stress tolerance while maintaining favorable agronomic and yield traits.

In Arabidopsis, unsaturated aliphatic components account for about 60% of total stem cutin, with C18:2 dioic acid being the predominant unsaturated component (Bonaventure et al., 2004). It is known that FATTY ACID DESATURASE2 (FAD2) affected cutin monomer composition, and the *fad2* mutation causes a 2-fold reduction in the levels of C18:2 dioic acids and a 3-fold increase in the levels of C18:1 dioic acids, suggesting that fatty acid desaturation reactions are fundamental to cutin biosynthesis (Bonaventure et al., 2004). The *fad2* mutant exhibited hypersensitivity to tunicamycin, a chemical inducer of ER stress (Nguyen et al., 2019). These results suggest that cutin composition and membrane lipid polyunsaturation are involved in ER stress tolerance in Arabidopsis. Very recently, we isolated a mutant showing an acquired osmotolerance–defective phenotype (*aod2*), which was impaired not only in acquired osmotolerance, but also in osmo-shock, salt-shock, and long-term heat tolerances compared with the WT (Fukuda et al., 2022). The causal gene of the *aod2* phenotype was identical to *CER10*, which encodes an enoyl-coenzyme A reductase that is involved in the elongation reactions of VLCFAs. In *aod2*, the amounts of major wax components were decreased, and ER stress mediated by bZIP60 was enhanced under osmotic stress compared with those in Bu-5, indicating that *CER10* plays a crucial role in these abiotic stress tolerances *via* VLCFA metabolism, which is required for cuticular wax synthesis and endocytic membrane trafficking (Fukuda et al., 2022).

## Conclusion

*KLU* overexpression improved not only acquired osmotolerance, but also osmo-shock, salt-shock, oxidative, S-heat, and L-heat tolerances of Arabidopsis. *KLU* overexpression led to high accumulation of cutin monomers and VLCFAs, resulting in increased epidermal cuticular wax load. Moreover, the *AtKLU*ox plants suppressed ER stress induced by osmotic and heat stresses. Taken together, our results suggest that the *AtKLU*ox plants exhibited tolerances to a wide range of abiotic stresses by suppressing water loss and ER stress through high accumulation of cutin monomers, VLCFA derivatives, and epidermal cuticular wax. KLU may be able to use long-chain fatty acids as substrates, although further biochemical and structural analyses are needed.

## Data Availability Statement

The original contributions presented in the study are included in the article/Supplementary Material, further inquiries can be directed to the corresponding author.

## Author Contributions

TK, MY, and TT initiated, conceived, and coordinated the project. JN performed FOX hunting. MY generated *AtKLU*-overexpressing Arabidopsis plants. TK and MY performed physiological analyses. TK observed epicuticular wax crystals by scanning electron microscopy with YY. YO and TK determined the wax and cutin components. AN performed protein structure prediction with AlphaFold2. TT wrote the manuscript with assistance from IY and YS. All authors contributed to the article and approved the submitted version.

## Funding

This work was supported by KAKENHI grants from the Ministry of Education, Culture, Sports, Science and Technology of Japan (19H03092 and 21H05668 to TT) and by the Asahi Glass Foundation, Japan (to TT).

## Conflict of Interest

The authors declare that the research was conducted in the absence of any commercial or financial relationships that could be construed as a potential conflict of interest.

## Publisher’s Note

All claims expressed in this article are solely those of the authors and do not necessarily represent those of their affiliated organizations, or those of the publisher, the editors and the reviewers. Any product that may be evaluated in this article, or claim that may be made by its manufacturer, is not guaranteed or endorsed by the publisher.

## Supplementary Material

Supplementary Figure S1 Acquired osmotolerance of *EsKLU*ox and *AtKLU*ox plants. Two-week-old WT, *EsKLU*ox and *AtKLU*ox plants on 750 mM sorbitol for 15 days following treatment on 100 mM NaCl. #2, #3, #4, #5 and #6 are independent *EsKLU*ox lines, and #1, #2, #4 and #5 are independent *AtKLU*ox lines.

Supplementary Figure S2 Morphology of *EsKLU*ox and *AtKLU*ox plants. **(A)** Examples of 7-week-old WT (upper left), *FOX351/EsKLU*ox #4 (upper right), and *AtKLU*ox (bottom) plants grown in soil under normal conditions. **(B)** Representative phenotypes of WT, *AtKLU*ox #4, *AtKLU*ox #5 plants at different stages. The stems were removed at 56 days stage.

Supplementary Figure S3 Anthocyanin content of *AtKLU*ox plants. Anthocyanin content of WT and *AtKLU*ox plants. Two-week-old WT and *AtKLU*ox plants grown on agar plates were used as the samples. Differences between WT and *AtKLU*ox plants were analyzed by Student’s *t*-test. ^*^*P* < 0.05; ^***^*P* < 0.001.

Supplementary Figure S4 Magnified view of cuticular wax contents described in Figure 5C. Aalkanes, ketones, aldehydes, and primary alcohols in WT and *AtKLU*ox plants. Data represent means ± SE, *n* = 6. Within each compound type and chain length, values marked with the same letter are not significantly different based on one-way ANOVA and Tukey’s test, *P* < 0.05.

Supplementary Table S1 Primers used for cloning.

Supplementary Table S1 Primer sets used for quantitative real-time polymerase chain reaction.
