# Supplementary figures and images for: KLU/CYP78A5, a Cytochrome P450 Monooxygenase Identified via Fox Hunting, Contributes to Cuticle Biosynthesis and Improves Various Abiotic Stress Tolerances
Source: Front Plant Sci. 2022 Jun 23;13:904121. doi: 10.3389/fpls.2022.904121 (PMC9262146; doi:10.3389/fpls.2022.904121)

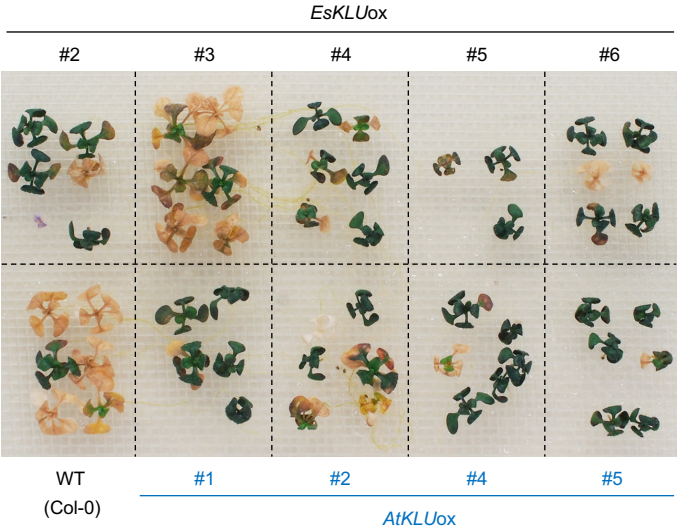

A

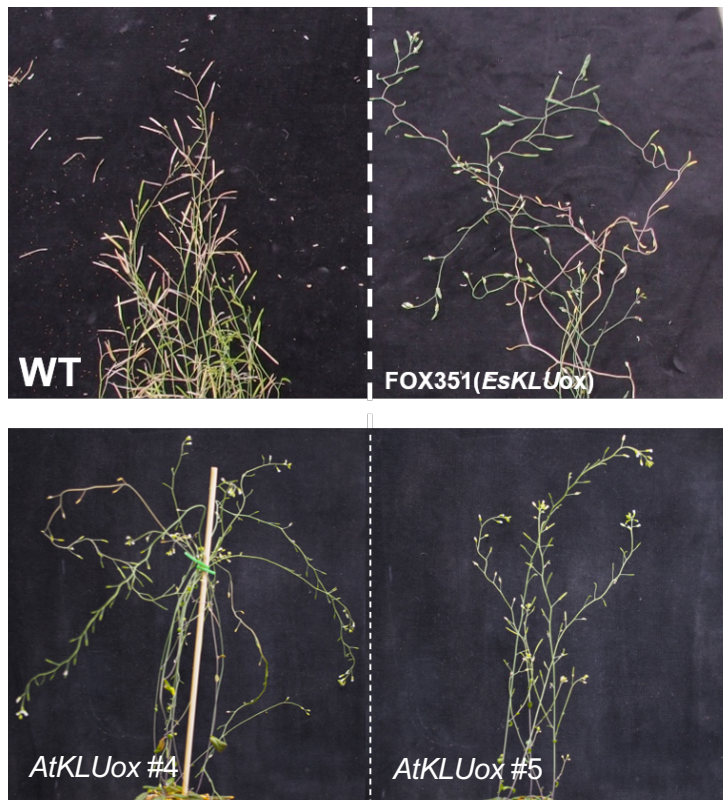

B

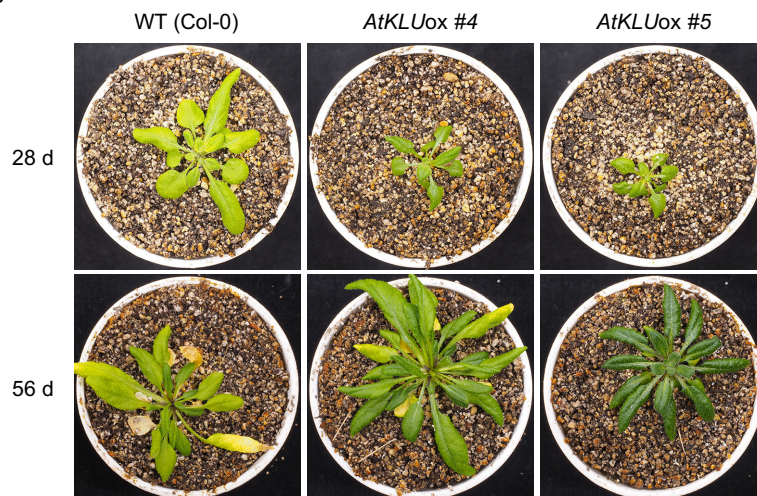

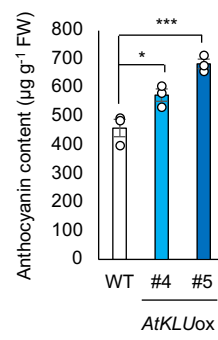

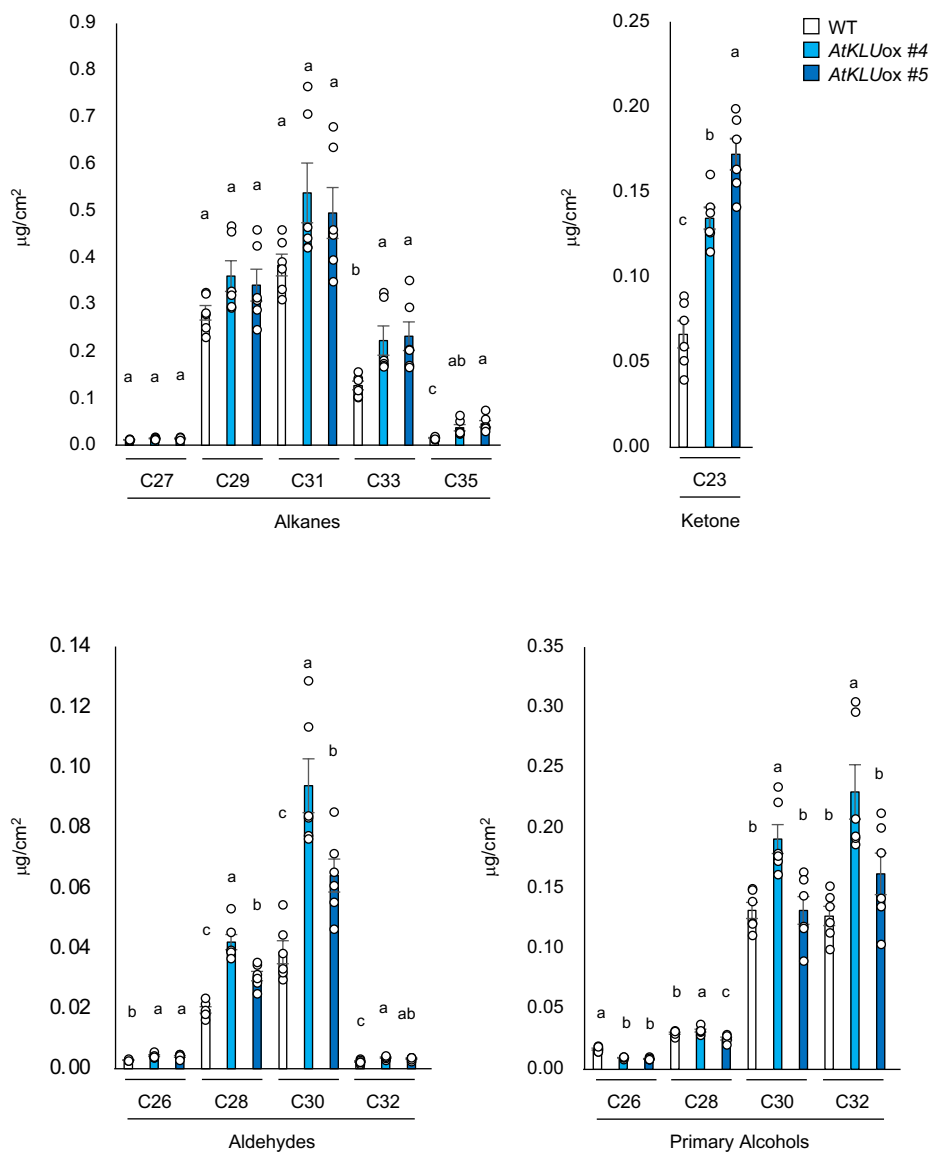

Supplement: Supplementary Figure S1 — Acquired osmotolerance of EsKLUox and AtKLUox plants. Two-week-old WT, EsKLUox and AtKLUox plants on 750 mM sorbitol for 15 days following treatment on 100 mM NaCl. #2, #3, #4, #5 and #6 are independent EsKLUox lines, and #1, #2, #4 and #5 are independent AtKLUox lines. [file Data_Sheet_2.PDF]
