# Supplementary material for: KLU/CYP78A5, a Cytochrome P450 Monooxygenase Identified via Fox Hunting, Contributes to Cuticle Biosynthesis and Improves Various Abiotic Stress Tolerances
Source: Front Plant Sci. 2022 Jun 23;13:904121. doi: 10.3389/fpls.2022.904121 (PMC9262146; doi:10.3389/fpls.2022.904121)
Supplement: Supplementary Table S1 — Primers used for cloning. [file Data_Sheet_1.PDF]

Supplementary Table S1. Primers used for cloning

Primer name	Sequence (5' → 3')
pGH AtCYP78A5/KLU F	cactgttgatacatatgtctccggaagcttacgt
pGH AtCYP78A5/KLU R	attcagaattgtcgatcaagcgaaaccaacattcct

Supplemental Table S2. Primer sets used for quantitative real-time polymerase chain reaction

Primer name	Sequence (5'→ 3')
Actin2 qRT F	ACCTTGCTGGACGTGACCTTACTGAT
Actin2 qRT R	GTTGTCTCGTGGATTCCAGCAGCTT
RAB18 qRT F	CCGTTAAGCTTCGAACAATCGTGT
RAB18 qRT R	CAACACACATCGCAGGACGTACA
RD29A qRT F	TGGATCTGAAGAACGAATCTGATATC
RD29A qRT R	GGTCTTCCCTTCGCCAGAA
KIN1 qRT F	GGAAGGCATTCTTGTTGGTCTCTG
KIN1 qRT R	GCCCACATCTCTTCTCATCATCAC
COR15A qRT F	GAGGCATTAGCAGATGGTGAGA
COR15A qRT R	CCACATACGCCGCAGCTTTCT
bZIP60 qRT F	CGGTTTCAGATTTCATAGCGGA
bZIP60 qRT R	GAATTCTCCTTCCCGGAATCAT
SAR1A qRT F	TTCACATGCTCAAAGATGAGAGA
SAR1A qRT R	TGCCAATGCTAAGTTCCTCAG
PDI9 qRT F	GCCCTGTTGAAGTGACTGAACT
PDI9 qRT R	TGTCAGGTAAGAAAGAGATAAAGCAA
SEC31A qRT F	GATTTCTCTGTATGCAGAACCTGA
SEC31A qRT R	GATTTCTCTTGATATGGATTGGAAA
HSP70 qRT F	AGGAGCTCGAGTCTCTTTGC
HSP70 qRT R	AGGTGTGTCGTCATCCATTC
HSP17.6 qRT F	GGTGAGTGGCAAAAGACAGA
HSP17.6 qRT R	AAACTTCCCCATCCTCCTCCTCT
HsfA2 qRT F	GTGTTGAGGTTGGGCAATACG
HsfA2 qRT R	TTGCTGTTGCCTCAACCTAACTAC
Es/AtCYP78A5/KLU qRT F	ACCTTGCTGGACGTGACTTCGTTGA
Es/AtCYP78A5/KLU qRT R	GTTGTCTCGTGGATTCCAGCAGCTT
